# 3D anatomical and perfusion MRI for longitudinal evaluation of biomaterials for bone regeneration of femoral bone defect in rats

**DOI:** 10.1038/s41598-017-06258-0

**Published:** 2017-07-21

**Authors:** Emeline J. Ribot, Clement Tournier, Rachida Aid-Launais, Neha Koonjoo, Hugo Oliveira, Aurelien J. Trotier, Sylvie Rey, Didier Wecker, Didier Letourneur, Joelle Amedee Vilamitjana, Sylvain Miraux

**Affiliations:** 1 Centre de Résonance Magnétique des Systèmes Biologiques, UMR5536, CNRS, Univ. Bordeaux, F-33076 Bordeaux, France; 2 Inserm U1026, Univ. Bordeaux, Tissue Bioengineering, U1026, F-33076 Bordeaux, France; 3 Inserm U1148, LVTS, X. Bichat Hospital, University Paris Diderot F-75018 Paris, Institut Galilée, University Paris 13, 93430 Villetaneuse, France; 4 Bruker Biospin MRI GMBH, Ettlingen, Germany

## Abstract

Magnetic Resonance Imaging (MRI) appears as a good surrogate to Computed Tomography (CT) scan as it does not involve radiation. In this context, a 3D anatomical and perfusion MR imaging protocol was developed to follow the evolution of bone regeneration and the neo-vascularization in femoral bone defects in rats. For this, three different biomaterials based on Pullulan-Dextran and containing either Fucoidan or HydroxyApatite or both were implanted. *In vivo* MRI, *ex vivo* micro-CT and histology were performed 1, 3 and 5 weeks after implantation. The high spatially resolved (156 × 182 × 195 µm) anatomical images showed a high contrast from the defects filled with biomaterials that decreased over time due to bone formation. The 3D Dynamic Contrast Enhanced (DCE) imaging with high temporal resolution (1 image/19 s) enabled to detect a modification in the Area-Under-The-Gadolinium-Curve over the weeks post implantation. The high sensitivity of MRI enabled to distinguish which biomaterial was the least efficient for bone regeneration, which was confirmed by micro-CT images and by a lower vessel density observed by histology. In conclusion, the methodology developed here highlights the efficiency of longitudinal MRI for tissue engineering as a routine small animal exam.

## Introduction

To evaluate the outcomes of new biomaterials on bone regeneration and vascularization and compare their *in vivo* efficiencies, non-invasive imaging techniques are required. The conventional imaging method for the analysis of bone repair is based on X-ray. Micro computed tomography (micro-CT) is an important tool to acquire quantitative 3D data for *in vivo* biological studies^1^. The main advantages of the micro-CT lie in the high-spatial resolution, sensitivity to skeleton, and a low cost. However, its use is limited by the relatively poor contrast of soft tissue and by the radiation damage in patients or in experimental models. Furthermore, to our knowledge, only anatomical information can be obtained *in vivo* within the bone, whereas functional data are essential to characterize the newly formed tissue^2, 3^.

On the other hand, Magnetic Resonance Imaging (MRI) appears as a non-invasive and non-ionizing technique well suited to *in vivo* longitudinal evaluation of tissue repair following a grafting procedure. This technique is a good candidate, both to visualize biomaterials within bones and to obtain functional data on the angiogenesis within the defect. Indeed, cortical bone is a tissue with a well-known very short transversal relaxation time (T2), whereas biomaterials during early period after implantation possess very long T2^4^. The balanced Steady-State Free Precession (bSSFP) sequence using a water frequency-selective (noted WS) binomial pulse was therefore employed since its contrast is related to T2/T1 of tissues, enabling the detection of defects filled with biomaterials with hyperintense signal. Other interesting features are the ability to acquire 3D images with high Signal-to-Noise Ratio (noted SNR) in short acquisition time. This is an important asset when cohorts of animals have to be scanned over time.

In parallel, to obtain information on the functionality of newly-formed blood vessels, gadolinium-based contrast agents can be injected in the systemic circulation. The information obtained from Dynamic Contrast Enhancement (DCE) T1-weighted MR images have also been used to monitor angiogenesis over time within implanted constructs. However, most of the studies were performed in rabbits^5–7^, large animals^8–11^ and patients^12^, so as to limit voxel size and consequently shorten acquisition time. The spatial resolution has to be improved in small animals, like rats, as the structures are smaller. As an example, the femoral bone has a diameter of 3 mm in 8-week old rats. Nevertheless, small animals are very convenient to study critical-sized bone defects and then develop diagnosis techniques and innovative therapies.

In order to apply such studies onto small animals, high spatial and temporal resolutions are required. Indeed, heterogeneities within the biomaterials need to be assessed to evaluate the full recovery of the defect. High sensitivity of the MR images is also necessary in order to evaluate variabilities between animals and to measure the efficiencies of several constructs depending on their different compositions.

Consequently, the objective of this work was to develop a non-invasive MR imaging protocol that can be applied on an experimental bone defect model in rats, to repetitively survey osteointegration of three biomaterials in bony sites. Indeed, our previous work has shown the osteoconductivity and osteoinductivity of a new macroporous scaffold composed of natural hydrophilic polysaccharides supplemented with hydroxyapatite (Matrix-HA)^4, 13^. This polysaccharide–based matrix can be supplemented with another natural sulfated polysaccharide extracted from brown algae, fucoidan(noted Fuco thereafter), having an heparin-like structure and reported to modulate the heparin-binding angiogenic growth factor (FGF-2) activity and to induce angiogenesis *in vitro* ^14–16^. Three different matrices were thus investigated for their properties in bone repair and vascularization: Matrix-Fuco, Matrix-HA, Matrix-HA-Fuco. Both anatomical and functional MRI studies were performed to fully characterize the new tissue in 3D within the bone defect. In parallel, *ex vivo* micro-CT and histology were also performed, in order to evaluate and quantify bone formation and compare the data from the different imaging modalities.

## Results

### Anatomical information of the newly regenerated tissue within the bone defect

#### *In vivo* MRI analysis

**Figure 1:**
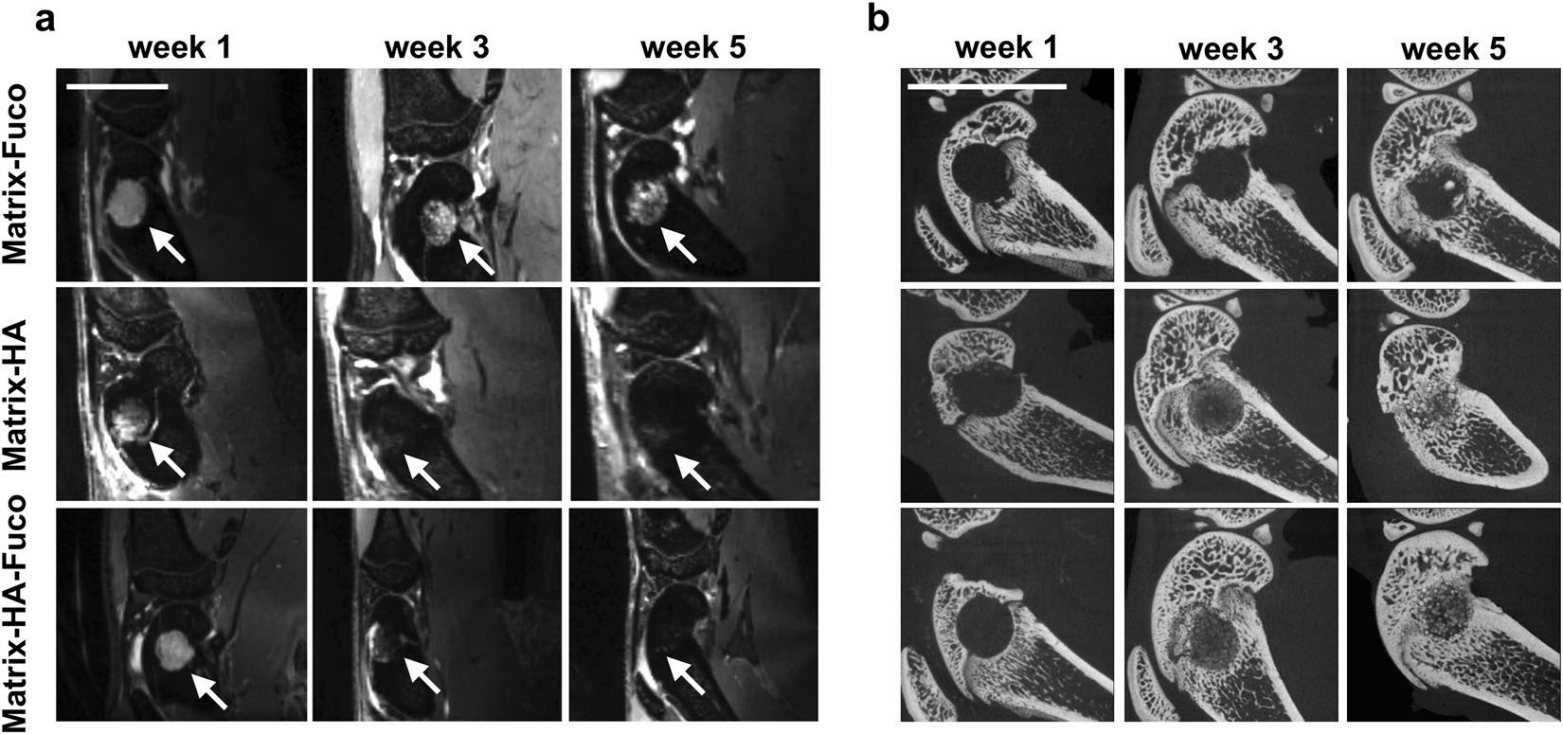
(**a**) 3D WS-bSSFP *in vivo* representative images and (**b**) representative 2D pictures of the median region of the *ex vivo* Micro-CT 3D reconstructed femoral condyles, 1, 3 and 5 weeks after implantation of Matrix-Fuco, Matrix-HA, and Matrix-HA-Fuco matrices (arrows). The number of samples used for both techniques is indicated in Tables 1 and 2, respectively. The MRI signal from defects filled with Matrix-HA and Matrix-HA-Fuco disappeared 5 weeks after implantation. At week 5, mineralization was visible within the defect site for these latter conditions. Scale bar represents 1 cm.

The WS-bSSFP sequence was performed to obtain anatomical information after implantation of biomaterials in the rats. Contrarily to healthy bones that appear hypo-intense, all the defects filled with biomaterials were easily detected as hyper-intense areas within the bone marrow one week after the implantation (arrow in Figs 1a and S1 and S2). For the defects filled with Matrix-HA and Matrix-HA-Fuco biomaterials, the hyper-intense signal disappeared over time, whereas the signal of the defect containing Matrix-Fuco remained even 5 weeks after implantation (Fig. 1a).

**Figure 2:**
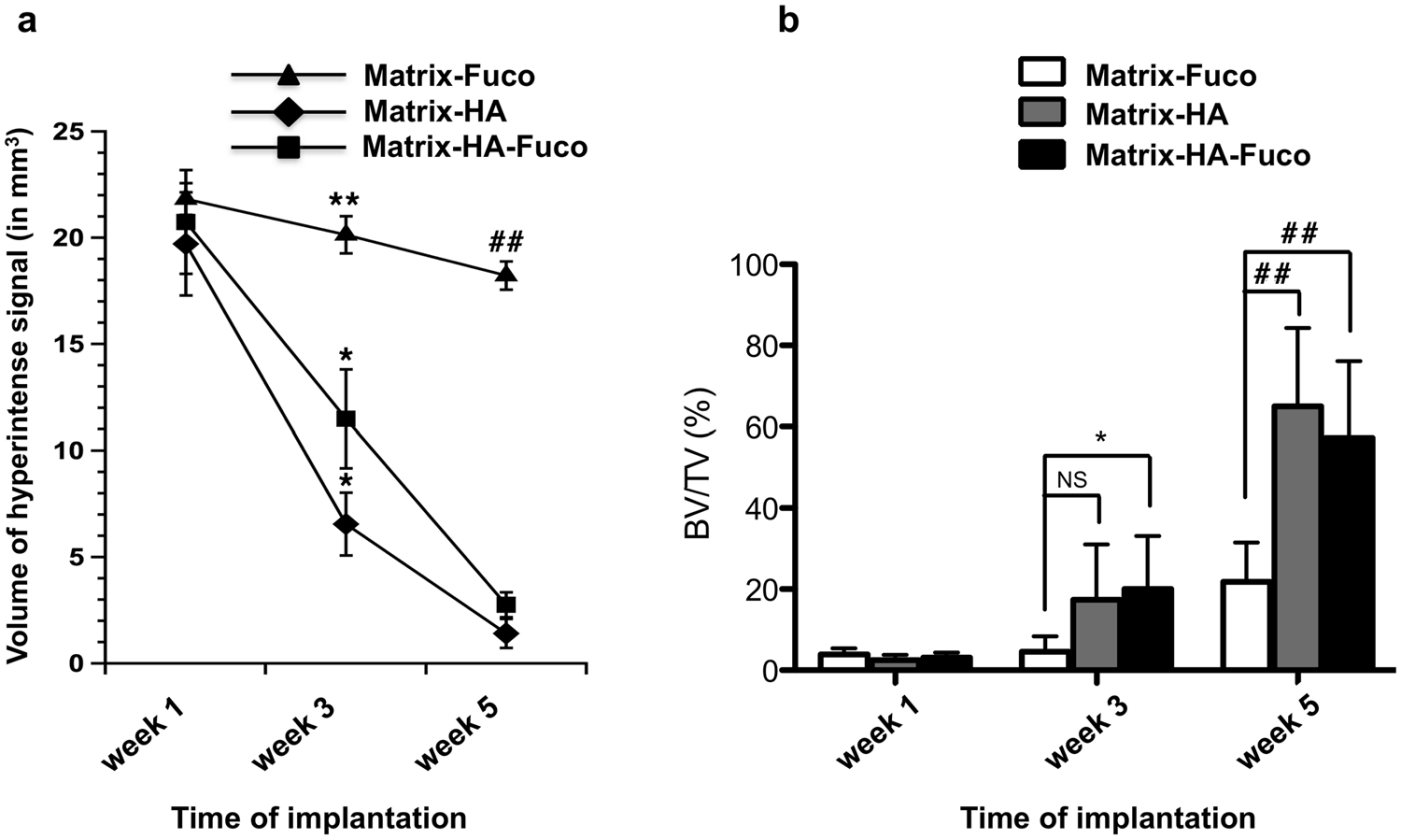
(**a**) Volume of the hyper-intense signal measured on the *in vivo* 3D WS-bSSFP images within the bone defects filled with the 3 biomaterials over weeks 1, 3 and 5. (**b**) BV/TV measurements from the *ex vivo* Micro-CT scans analysis. NS: Non Significant differences. Significant differences between biomaterials at each week are indicated with *p < 0.05 and **p ≤ 0.01 for week 3 and ^##^p ≤ 0.01 for week 5. Of note, the MRI volumes measured in Matrix-HA-Fuco filled defects were significantly different to each other at every time point. The MRI volumes measured in Matrix-HA filled defects at week 1 was significantly different to the ones obtained at week 3 and week 5. Similarly, the MRI volumes measured in Matrix-Fuco filled defects at week 5 was significantly different to the one obtained at week 1.

The volume of the hyper-intense signal measured in the Matrix-HA rats decreased by 66.7% and 92.8% at week 3 and 5, respectively (Fig. 2a). Similar results were obtained for the Matrix-HA-Fuco biomaterial (57.3% and 91.7% at week 3 and 5, respectively). While no statistical difference between these two materials has been observed at week 5, the volume of the hyper-intense signals in the rats implanted with the Matrix-Fuco material had a different behavior as it remained similar over time (Fig. 2a).

#### *Ex vivo* Micro-CT analysis

One week after implantation, micro-CT images show a high signal coming from healthy bone and a lack of signal within the defect (Supplementary Fig. S1). None of the biomaterials could be detected on CT images at any time point.

In parallel to the MRI measurements, micro-CT images revealed, with time of implantation from week 1 to week 5, numerous spots of mineralized tissue inside the defects filled with Matrix-HA and Matrix-HA-Fuco, especially at week 5, whereas Matrix-Fuco filled defects only partially evidenced few spots of mineralized tissue (Fig. 1b). These observations were supported by the quantification of the Bone Volume to Total Volume (BV/TV) in the three groups of matrices (Fig. 2b). Mineralization increased over time for the three groups of matrices. Significant differences of tissue mineralization was observed at week 3 and 5 between the Matrix-HA and Matrix-HA-Fuco groups, compared to Matrix-Fuco group.

#### *Ex vivo* Histological analysis

**Figure 3:**
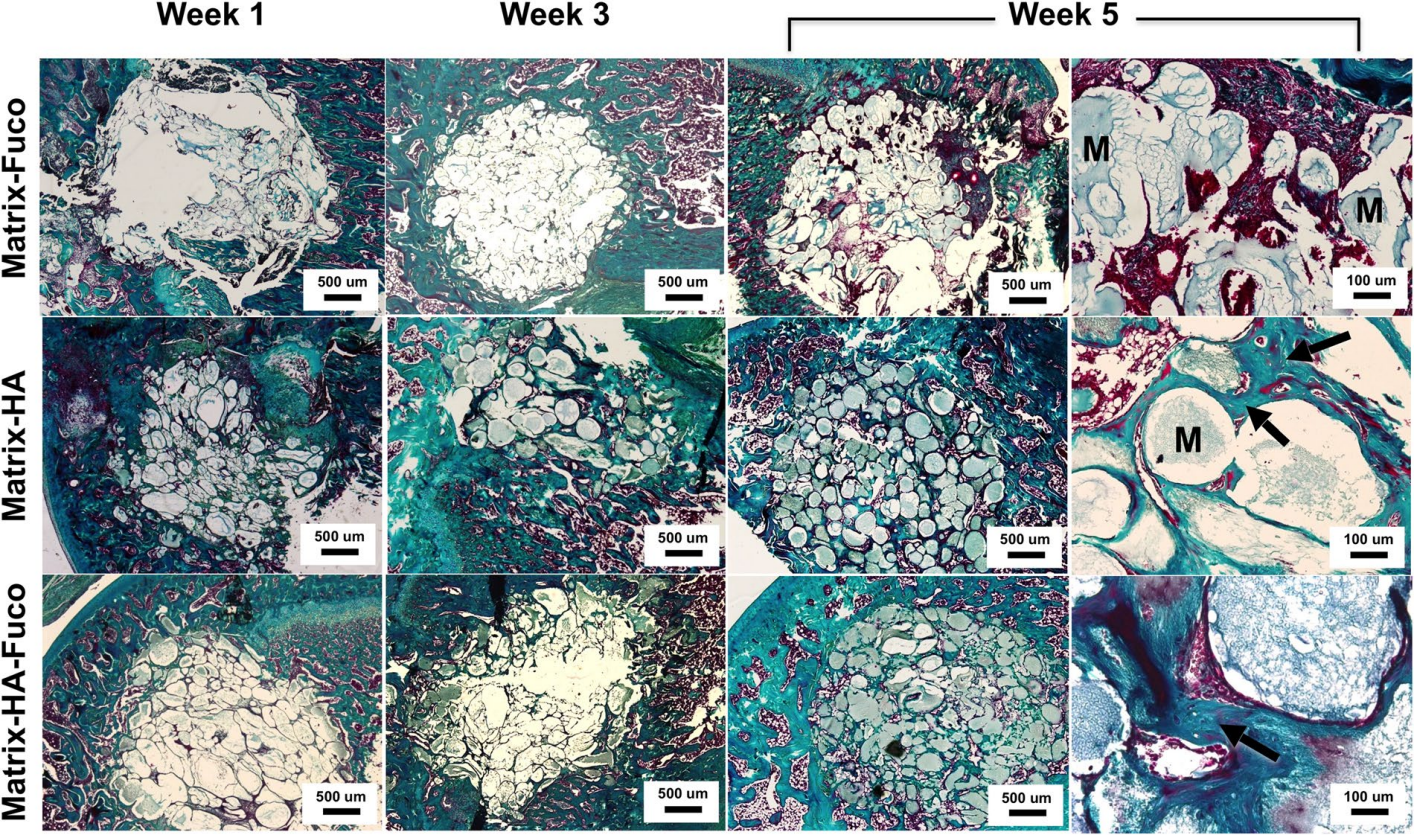
Representative images of histological staining of the bone defects that received the biomaterials at week 1, 3 and 5. Decalcified sections were stained with Masson’s Trichrome. Increase of osteoid, mineralized bone tissue and osteocytes are visible over time (arrows on the magnification images) around the beads of Matrix-HA and Matrix-HA-Fuco, especially at week 5. M: Matrix.

The qualitative and quantitative analyses obtained by MRI and micro-CT were confirmed by histological data and Masson’s Trichrome staining (Figs 3 and S1). We observed osteoid tissue and mineralized bone tissue around the beads of matrices of both Matrix-HA and Matrix-HA-Fuco, mainly at week 3 and evidenced at week 5. For Matrix-Fuco group, few osteoid tissues were observed in some areas of the defect (Fig. 3) mainly at week 5, that correlated with the spots of mineralized tissue observed by micro-CT at the same time point (Fig. 3).

**Figure 4:**
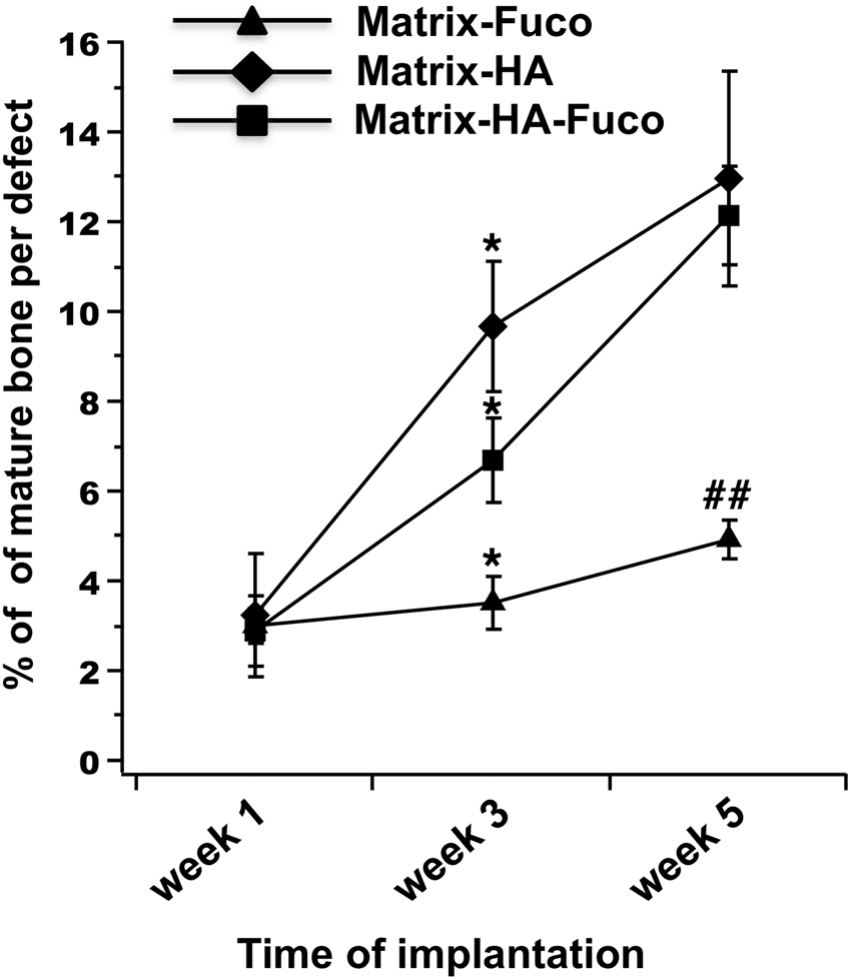
Percentage of bone formation within the implanted matrices over time of implantation. The quantification was performed from Trichrome Masson staining slides (n = 3) on the defects filled with the three groups of biomaterials over weeks 1, 3 and 5. Significant differences between biomaterials at each week are indicated with *p < 0.05 for week 3 and ^##^p ≤ 0.01 for week 5. Of note, the percentage of bone formation obtained on the Matrix-HA-Fuco filled defects were significantly different to each other at each time point. The percentage obtained on the Matrix-HA filled defects at week 1 was significantly different to the ones obtained at week 3 and week 5. Similarly, the percentage obtained on the Matrix-Fuco filled defects at week 5 was significantly different to the ones obtained at week 1 and week 3.

The histomorphometry analysis demonstrated that the percentage of mature bone increased significantly over time of implantation when the Matrix-HA and Matrix-HA-Fuco were implanted into the defects (Fig. 4). Five weeks after implantation, these two biomaterials enabled to similarly promote bone formation into the defects.

However, when the Matrix-Fuco biomaterials were implanted into the defect, the amount of mature bone remained similar over time, even though bone tissue started to regenerate after 5 weeks (Fig. 4). This last result is in agreement with the timeline of bone regeneration within an empty defect, as already shown by Schlaubitz S *et al*.^4^.

### Perfusion information of the newly regenerated tissue within the bone defect

#### *In vivo* DCE information

**Figure 5:**
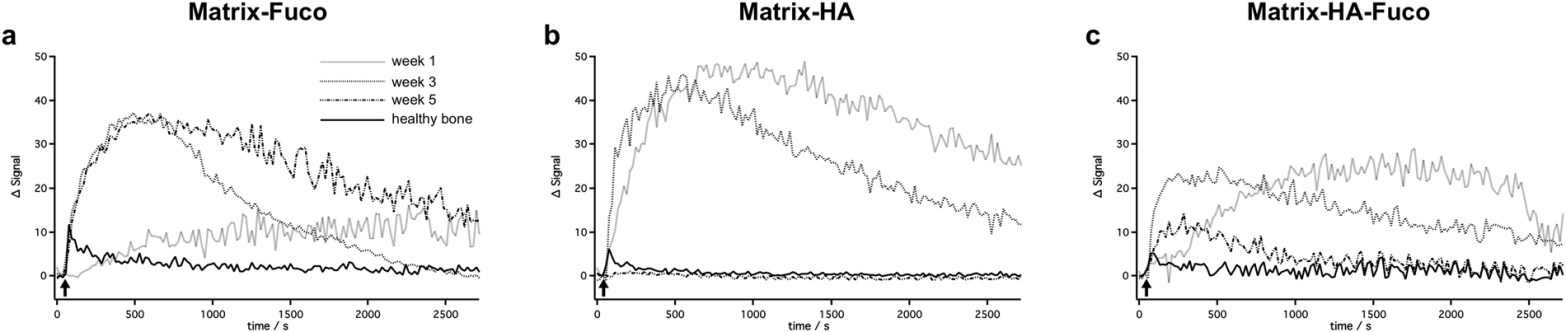
Representative MR ΔSignal (subtracted from the signal measured before injection) over time after the intravenous injection of Gd-DOTA (black arrow) at week 1, 3 and 5 after implantation of Matrix-Fuco (**a**), Matrix-HA (**b**), and Matrix-HA-Fuco (**c**) matrices.

3D T1-weighted MRI was performed with high spatial and temporal resolutions in order to follow the rapid biodistribution of the Gd-DOTA in the rat bone defects after its intravenous injection. One week after the implantation of the matrices, the subsequent Gd-DOTA positive signal was detected, starting from the periphery of the defect to slowly diffuse to the center. The signal in the core of the defect gradually increased (Fig. 5). Forty-five minutes after the Gd-DOTA injection, the signals of the defect were still higher than before injection. This pattern was detected for all the defects. Over the weeks after the implantation, the contrast agent reached the center of the defect faster, and was cleared out over the 45 min course follow-up. The signal curve measured at 5-week post-implantation was similar to the one measured in the healthy bone for the Matrix-HA and Matrix-HA-Fuco materials. However, for the Matrix-Fuco materials, even at 5 weeks after implantation, the curve of the signal was still very different from the one of healthy bone.

**Figure 6:**
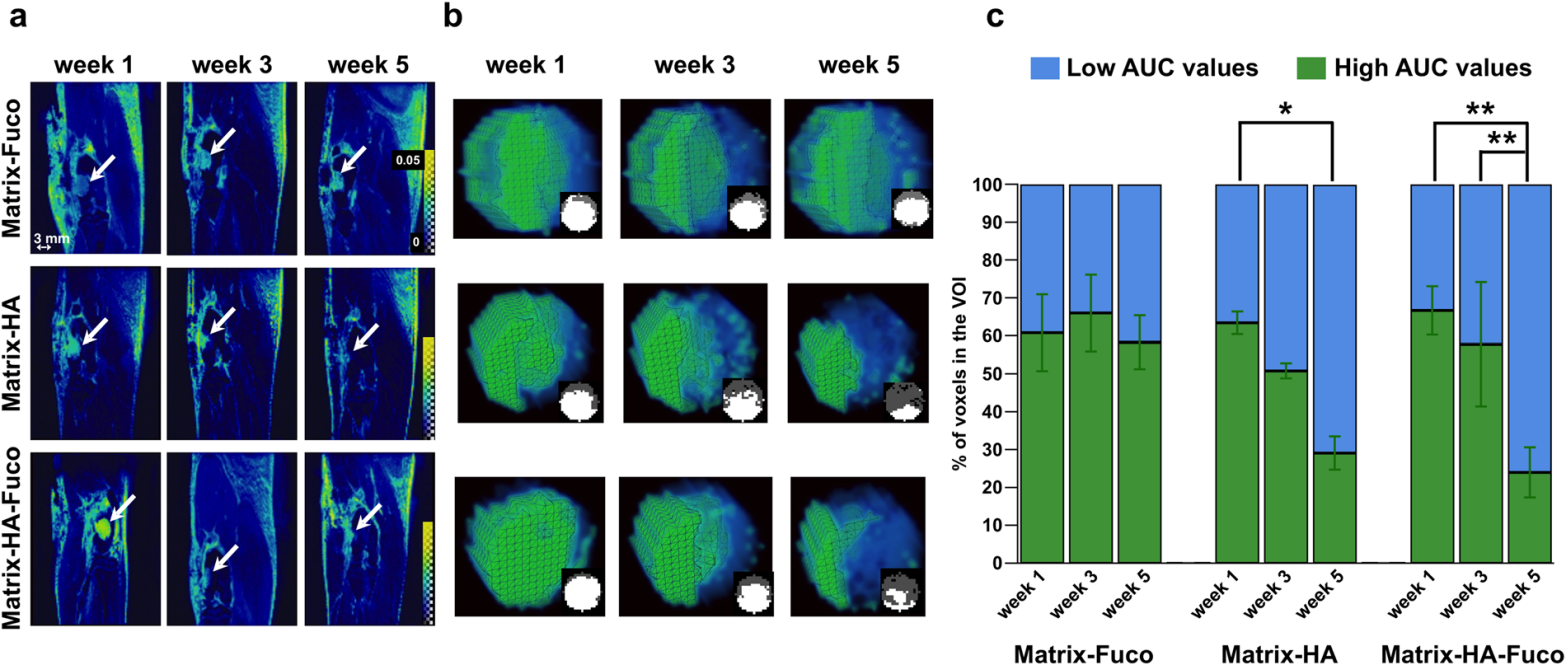
(**a**) 3D parametric AUC maps of the bone defects that received the biomaterials. A slice showing each defect at each time point was extracted from the 3D maps. For each biomaterial group, the same rat is displayed over weeks 1, 3 and 5. The white arrows show the defect. The color scale represents the AUC values between 0 and 0.05. (**b**) 3D rendering of each biomaterial defect extracted and magnified at weeks 1, 3 and 5 post-implantation. Voxels with high AUC values are colored in green and voxels with low AUC values are colored in blue. The small inserts are 2D slices extracted from the 3D AUC maps, located at the center of the defect. The amount of fast-perfused voxels (with low AUC) increased over time in the Matrix-HA and Matrix-HA-Fuco filled defects. In contrast, no change with time was measured in the defects filled with Matrix-Fuco biomaterial. (**c**) Quantitative analysis of the percentage of pixels with high (green) and low (blue) AUC from the extracted Volume-of-Interest (VOI) for the 3 different biomaterials at weeks 1, 3 and 5. Values are means ± Standard Deviations. Significant differences between each week are indicated with *p < 0.05 and **p ≤ 0.01. Of note, the percentage obtained on the Matrix-HA-Fuco filled defects at week 5 was significantly different to the ones obtained at week 1 and week 3. Similarly, the percentage obtained on the Matrix-HA filled defects at week 1 was significantly different to the ones obtained at week 5. On the contrary, the percentages obtained on the Matrix-Fuco filled were not significantly different at any time point.

To quantify these evolutions, the AUC over 45 minutes was measured within each voxel of the 3D T1-weighted MR images (Fig. 6a). Within a defect, two populations of voxels were detected depending on their AUC values. High AUC reflected a long remain time of the Gd-DOTA inside a voxel. On the contrary, low AUC reflected a fast wash-in/wash-out of the contrast agents in a voxel, similarly to the evolution of Gd-DOTA into the healthy bone tissue (Fig. 5). Due to the pixel-wise analyses, the amount of each population was quantified over time (Fig. 6b,c). The number of voxels with low AUC kept increasing over time, while the voxels with high AUC decreased in the Matrix-HA and Matrix-HA-Fuco filled defects. Indeed, the amount of voxels with low AUC corresponded to 49.2% and 70.8% at week 3 and 5, and 42.2% and 76% at week 3 and 5, for the Matrix-HA and Matrix-HA-Fuco groups, respectively. In addition, the 3D AUC maps demonstrated that the periphery of the defects mostly contained voxels with low AUC, whereas high AUC was mainly measured in the core of the defects. Furthermore, the thickness of this rim containing voxels with low AUC increased over time by 1.7 ± 0.2 mm and 2.2 ± 0.2 mm from week 1 to week 5, for the Matrix-HA-Fuco and Matrix-HA groups, respectively. On the contrary, the amount of voxels with low AUC remained similar over time for the Matrix-Fuco group (representing around 40% of the total amount of voxels within the defects) (Fig. 6b).

#### Immunohistochemistry

**Figure 7:**
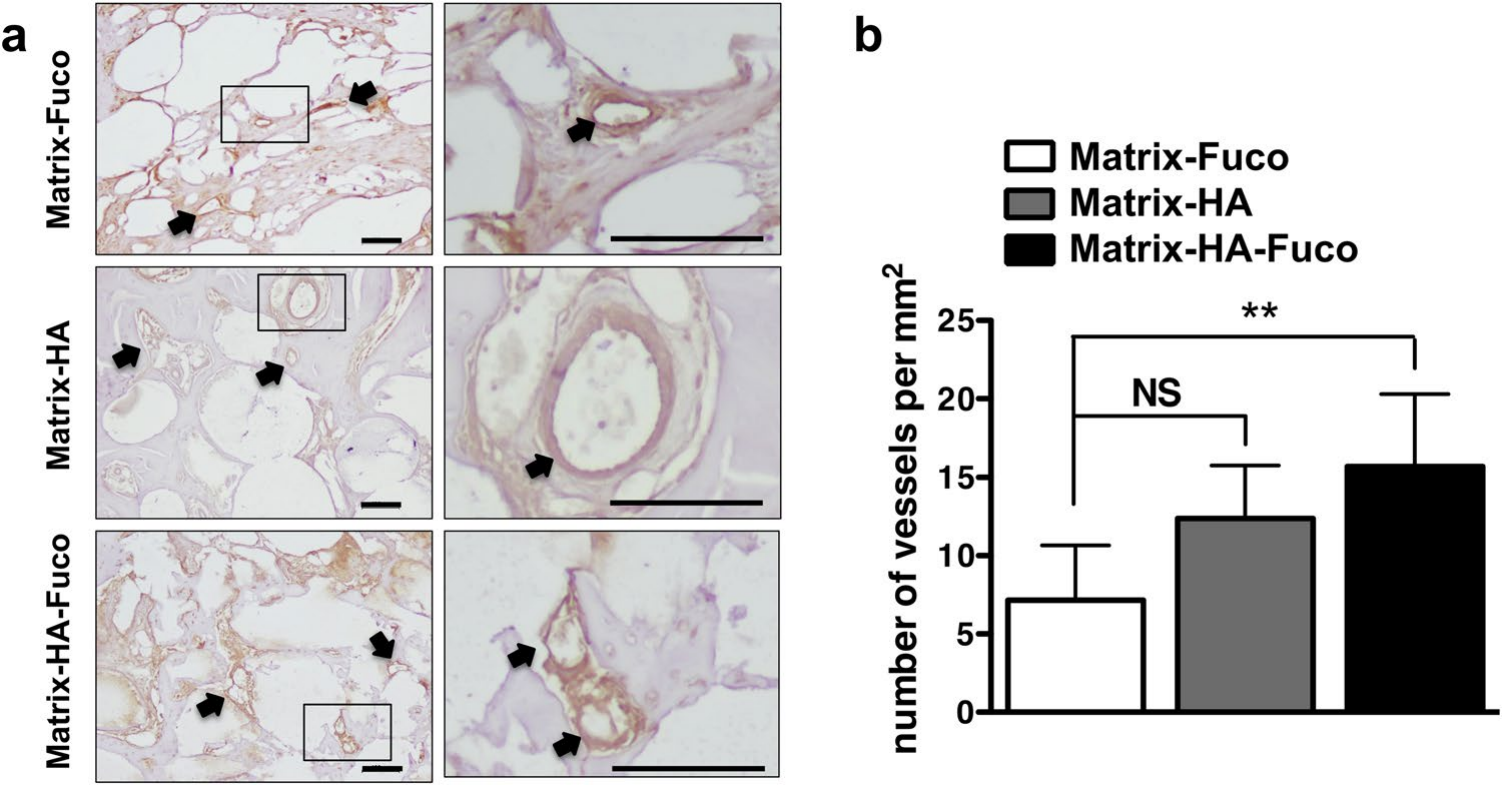
Immunohistochemistry evaluation of CD31 expression. (**a**) Representative images of CD31 staining of vessels in the three groups of implanted matrices (scale bar = 100 µm). (**b**) Quantification of vessel density inside the implanted matrices at 5 weeks post implantation (n = 6; Average ± SD). NS and **denote Non Significant and p < 0.01, respectively.

Immunohistochemistry of CD31 blood vessel staining was performed at sacrifice after 5 weeks of implantation (Fig. 7a). As indicated by the black arrows (Fig. 7a), blood vessels could be found throughout the section and around the implanted material. As means to quantify the formation of blood vessels, we evaluated the number of vessels per mm^2^. As observed in Fig. 7b, a significant increase in terms of vessel density could be observed for the Matrix-HA-Fuco, in relation to Matrix-Fuco.

## Discussion

This study is the first study to combine *in vivo* MRI, *ex vivo* microCT and histology on the same small animals with bone defects over time. In addition, its originality also relies on the 3D anatomical and 3D perfusion MRI images that were obtained.

Small animal models are getting more interest in the field of tissue engineering especially to develop biomaterials for bone regeneration. Most of these studies are using Micro-CT imaging for longitudinal follow up. However, MRI appears to be a good surrogate due to its non-invasive properties, high contrast between tissues, the multi-parametric features in one exam and its translational potential combined with the development of musculo-skeletal MRI in clinics. Nevertheless, imaging of tissue engineering in rat models, especially for hindlimb bone regeneration, has been poorly investigated^4^. Indeed, 3D high spatial and temporal resolutions are needed to follow the bone regeneration in the bone femoris.

In this study, a high magnetic field combined with specific phased array surface coils were used to performed 3D anatomical and 3D perfusion imaging. Anatomical information were obtained by performing the 3D WS-bSSFP sequence. This T2-like high-contrast and high-SNR sequence was combined with a water-selective pulse to prevent high signal from adipose tissue, which is the main component of the bone marrow. This enabled to detect with high contrast any bone defects filled with biomaterials and to measure their signal longitudinally. Over time, this hyper-intense signal decreased, reflecting the formation of new bone tissue having very short T2 inside the defects. The sensitivity of this sequence enabled to demonstrate the efficiency with time of biomaterials in regenerating bone tissue.

Even though, no direct bone quantification could be performed on the MRI images, the measurement of the volume of the WS-bSSFP hyper-intense signal inversely correlated with the bone volume (BV/TV) calculated by micro-CT. Consequently, an indirect quantification of bone regeneration could be estimated using WS-bSSFP with no radiation involvement. In addition, the 3D WS-bSSFP sequence could be a good surrogate to micro-CT as it can be applied on small and large animals and on humans at high and clinical magnetic fields, even in the case of poor field homogeneity through the use of the Sum-Of-Square reconstruction^17^. The bSSFP sequence is already available on most of the clinical scanners, but is rarely performed in 3D due to banding artifacts. At magnetic fields lower than 4.7 T, only 1 or 2 bSSFP images are needed to suppress these artifacts due to a better field homogeneity. Consequently, acquisition time should be shorter than 2 minutes when applied on humans than in the current study, making the sequence of a huge interest for bone tissue engineering.

Anatomical information is essential in order to evaluate bone regeneration, but the functionality of the new tissue needs to be assessed. Angiogenesis is a crucial event that is necessary to generate viable bone tissue. Although iodine-based contrast agents can be injected intravenously to detect blood vessels^18^, micro-CT angiography is not trivial due to long exposures to radiations. Consequently, most of these studies are performed *ex-vivo*. On the contrary, perfusion MRI is a clinical routine technique and is often applied for small animal imaging using 2D T1-weighed MR sequences^19, 20^. In our case, 3D high spatial resolution was necessary in order to discriminate differences of perfusion within the defects. In addition, 3D high temporal images were performed to detect the rapid biodistribution of the contrast agent in the defects filled with the biomaterials. Contrarily to human imaging, the Arterial Input Function (AIF) was not measured in the current study, because of its poor accuracy in small animals. Consequently, the AUC was measured in order to discriminate perfusion states between different matrices-filled defects. Indeed, AUC already enabled to distinguish bladder constructs containing high and low amount of VEGF^6^.

The high resolution of the 3D DCE-MR images enabled to measure AUC heterogeneities within each defects. Indeed, the analysis of each entire defect showed that the AUC were smaller at the peripheries of the defects compared to large AUC that were located in voxels of the defect cores. These results were also obtained by Beaumont *et al*., where a calvaria model was studied using DCE-MRI in rabbits^21^. Like in our study, they measured two AUC values after Gd-DOTA injection depending on the core and the periphery of the materials. Imaging at high magnetic field (7 T here versus 1.5 T in Beaumont *et al*.) enabled to increase the spatial resolution (195 × 312 × 375 um versus 390 × 781 × 3000 um, respectively) without affecting SNR in order to study small defects (3.5 mm diameter in the rats versus 15 mm in the rabbits). Consequently, the current 3D MRI perfusion protocol would be of great interest to study and compare the neo-vascularisation in innovative bone substitutes.

In the field of bone tissue regeneration, the reconstruction of large volume defects remains challenging because of a lack of vascularization^22^. Several advanced techniques have been developed to overcome these limitations^23^ including production of tissue-engineered constructs that combine scaffolds with mesenchymal stems cells and/or endothelial progenitor cells^24, 25^, osteoinductive factors^26, 27^, or angiogenic growth factors^28^. However, their use is often associated with excessive inflammation, uncontrolled drug delivery, high costs^29^, and specific regulations and production for cell-based approaches^30^. Consequently, innovative cell-free and growth factor-free biomaterials able to promote both bone formation and vascularization are still expected. In this context, we have previously described osteoconductivity and osteoinductivity of a ready-to-use macroporous polysaccharide-based scaffold supplemented with hydroxyapatite (Matrix-HA)^4, 13^. Using the 3D *in vivo* MRI, we were able to perform here a longitudinal follow up of this matrix in bone repair and the comparison with new matrices supplemented with fucoidan, having heparin-like angiogenesis properties^14–16^. Our results indicated that the inclusion of fucoidan alone in the matrix was not sufficient to observe a significant repair process or neo-vascularization. Interestingly, *in vivo* 3D WS-bSSFP MRI clearly indicated a bone repair process using both the Matrix-HA and Matrix-HA-Fuco. In addition, the 3D MRI perfusion data correlated with vascularization observed by histology after sacrifice. In fact, one week after implantation of the biomaterials, a very slow accumulation of the contrast agent was observed from the periphery to the core of the biomaterials. This pattern, observed for the defects filled with the three matrices, was mostly due to not-yet vascularized defects. In that case, Gd-DOTA was supplied to the defects through already existing bone marrow vessels, and then slowly diffused inside the defects. After 5 weeks, Gd-DOTA reached the core of the defect more rapidly in the cases of Matrix-HA matrices. With these materials, the evolution of the signal was similar to the one measured in healthy bone (Fig. 4). This could be attributed to the formation of new blood vessels within these defects. This was confirmed by immunohistochemistry, showing a higher density of blood vessels in the Matrix-HA matrices-filled defects. This is in agreement with previous studies that demonstrated that AUC correlated well with micro-vascularity of a bladder acellular matrices^5–7^. Consequently, the high sensitivity of the 3D DCE-MRI technique enabled to characterize the perfusion heterogeneity between defects filled with biomaterials promoting or not neo-vascularization and within each defect, and also the evolution of the perfusion overtime.

The current protocol might be applicable to a wide range of biomaterials. Indeed, hydrogels^31^ or chitosan-based hydrogels^32^ has already been detected as hyper-intense signals on MR images. Consequently, it is expected that the same anatomical and perfusion images can be performed on these materials. Nevertheless, it might be less efficient when materials are more solid than the ones used in this study, for example the ones containing titanium. Indeed, as the MR signal would be severely affected, the contrast of the materials with the healthy bone would be largely reduced. This might prevent an accurate volume delineation. To circumvent this problem, specific MR sequences might be employed, like the ones using Ultra-short Echo Time (UTE).

To conclude, MRI has a tremendous potential for bone tissue engineering applications and the follow-up of the newly bone tissue regenerating within a defect. Both qualitative and quantitative data obtained by *ex vivo* Micro-CT, ex vivo histology and *in vivo* MRI indicated similar effects in terms of bone tissue regeneration, vascularization and perfusion. Due to the non-invasiveness of MRI, less animals were also used for imaging bone tissue formation compared to histological analysis. Taken together, MRI can be used as an alternative to micro-CT and histology/immunohistochemistry for preclinical bone tissue engineering studies, and is of great interest to non-invasively follow bone repair and vascularization in humans.

## Materials and Methods

### Preparation and characterization of polysaccharide-based porous beads

Three polysaccharide-based matrices were prepared using previously described methods^4, 13, 16^. Briefly, pullulan and dextran dissolved in water at a final concentration of 30% w/v (Pullulan, Mw 200,000, Hayashibara, Japan; Dextran, Mw 500,000, Pharmacosmos, Danmark) were chemical cross-linked with sodium trimetaphosphate (STMP, 25% (w/v), Sigma) under alkaline conditions. Fucoidan extracted from Fucus vesiculosus of medium molecular weight (Sigma-Aldrich, 10% w/w), Hydroxyapatite obtained by wet precipitation of a 0.6 M solution of phosphoric acid and a 1 M solution of calcium hydroxide^13^, or both were added to the polysaccharide solution to obtain Matrix-Fuco, Matrix-HA or Matrix-HA-Fuco. Sodium chloride (54% (w/w)) was also added as porogen agent to prepare porous scaffolds. Scaffolds were obtained after freeze-drying in the form of dried 300–500 µm beads as previously reported^4^.

### Animal models

Animal experiments were performed in accordance with the “Principles of Laboratory Animal Care” recommended by the National Society for Biomedical Research in France. Interventions were carried out in an accredited animal facility (authorization no. A33-063-917) at the University of Bordeaux, under authorization no. 5012032-A of the French Ministry of Agriculture and were approved by the Animal Ethic Committee of the University of Bordeaux.

Anesthesia was induced with a mixture of 4% isoflurane/Air 1–2 L/min (Baxter, Deerfield/IL, USA) and was maintained at 2% isoflurane/Air 0.8 L/min during the implantation. A stable body temperature was assured by the use of a heating device. Medial defects of 3.5 mm diameter and 4 mm depth were introduced in left and/or right femoral condyles of female Wistar RjHan rats at 10 weeks of age weighting 250–300 g (Janvier, Le Genest, France) using a dental microdrill (Thomas, France). Bone defects of about 38 mm^3^ were rinsed with physiological solution NaCl 0.9% (w/v). The defect was filled with dry beads of Matrix-Fuco, Matrix-HA, and Matrix-HA-Fuco, leaving enough time for the beads to hydrate with the blood invading the defect and, as a consequence, to increase slightly in size. The scaffolds remained solid enough to stay inside the defect. Therefore, no membrane was needed to avoid leakage of the biomaterial. Absorbable Vicryl sutures were performed on lateral muscles before closing the cutaneous plan with non-absorbable prolene sutures and suture clips. To assure analgesia, 0.3 mg/kg of buprenorphine (Vetergesic Multidose, Alstoe Veterinary, York, UK) was administered subcutaneously to each rat 30 min prior to the procedure as well as 24 hours afterwards. Additionally, animals were kept under free access to 0.2 mg/mL of Ibuprofen (Advil, Pfizer, Canada) 3 days prior to surgery and during 1 week after surgery through the drinking water.

The number of animals used for the MRI and micro-CT experiments are summarized in Tables 1 and 2, respectively. Animals were sacrificed by lethal injection (200 mg/animal) of sodium pentobarbital (Ceva, France).

**Table 1:** Number of samples used per group and per time-point for longitudinal MRI analyses.

Material	Matrix-Fuco	Matrix-HA	Matrix-HA-Fuco
Week 1	5	4	6
Week 3	5	4	6
Week 5	5	4	6

**Table 2:** Number of samples used per group and per time-point for *ex vivo* micro-CT analyses.

Material	Matrix-Fuco	Matrix-HA	Matrix-HA-Fuco
Week 1	8	9	15
Week 3	7	8	7
Week 5	9	7	8

### MRI study

A total of 15 rats were used for the MRI longitudinal study. These rats were implanted in only one limb and five implants were imaged per group of biomaterials (Table 1). Longitudinal *in vivo* MRI was performed on the same rats from each group, 1, 3 and 5 weeks post-implantation. The MR images were acquired on a horizontal 7 T magnet (Bruker, Biospec, Germany). The system was equipped with a 12 cm gradient insert capable of 660 mT/m maximum strength and 110 µs rise time. The rat knees were imaged using an emission 4-phased array surface coil. Rats were anesthetized with isoflurane (1.5% in air). The respiration rate was monitored using an air balloon place on top of the lungs (SA Instruments Inc., NY, USA).

### Anatomical 3D imaging

A 3D bSSFP sequence was used in which the usual radiofrequency pulse was replaced by a water frequency-selective binomial pulse (containing 5 hermite sub-pulses). Each pulse lasted 150 μs, the interpulse delay was set to 200 μs, the intensities of the pulses followed the schematic 1-2-3-2-1. The slice selection gradient was removed. The other sequence parameters were as follow: TE/TR = 1.9/5.1 ms; reception bandwidth = 75 kHz; FOV = 30 × 35 × 25; matrix = 192 × 192 × 128; 4 phase offsets; FA = 27°; 2 averages; Total acquisition time = 17 min. The 3D images were reconstructed after a “Sum-Of-Square” of the 4 offsets^33^.

### 3D DCE-MRI

DOTAREM (Guerbet, France) containing Gd-DOTA, a T1-contrast agent commonly used in clinics, was intravenously injected at each scanning session (400 µL of a dose of 100 mM). 3D T1-weighted images were acquired before and after the injection with the following parameters: FOV = 25 × 30 × 12; matrix = 128 × 96 × 32; TE/TR = 1.9/6.5 ms; flip angle = 30°; rBW = 75 kHz. One image was acquired in 19 s and the clearance of Gd-DOTA was followed over 45 min.

### Image Analysis

The volume of the hyperintense signal located within the defect and detected on the anatomical WS-bSSFP images was measured using the Amira software. For this purpose, a 3D region of interest (ROI) was manually drawn to englobe voxels with signal superior to the signal of the surrounding healthy bone (signal of 43.3 ± 11.3 a.u.).

The analyses of the DCE-MRI were performed on a pixel-wise basis using the Matlab software (Mathworks Inc., France). To minimize variabilities between animals, the signal to noise ratio (SNR) was normalized to the signal from the calf muscle and to the noise. For this purpose, independent ROIs were drawn in the muscle and outside of the animal’s tissue. Finally, a 3D cylinder-shaped mask was applied on the images acquired at week 1 in order to select the defect within the healthy bone (healthy bone represented 36.4% ± 2.9 of the total amount of the voxels within the mask). This mask was applied on the images acquired at week 1, 3 and 5 after implantation.

The area under the SNR curve for 45 min after the contrast agent injection (AUC) was computed for each pixel within the mask. Histograms representing the amount of voxels in function of the AUC values were created in order to manually set a threshold separating high versus low AUC values. Low AUC values were identified as similar to the AUC of healthy bone. The threshold was determined for every rat at each time point in order to counteract differences in arrival time of the contrast agent to the defects due to the DOTAREM manual injection.

### Micro-Computed Tomography study

18 rats were implanted in both sides (right and left femoral condyles) per group of matrices. The bones were extracted for *ex vivo* micro-CT at week 1, 3 and 5 after implantation into the condyles. Table 2 summarizes the number of samples used for each time point and for the three groups of matrices after the exclusion of the ones exhibiting﻿ spontaneous fractures after surgery.

The X-ray microtomographic equipment used in this study was a Quantum FX Caliper (Life Sciences, Perkin Elmer, Waltham, MA). The X-ray source was set at 90 V and 160 μA. Samples were scanned up to a Field of View of 10 mm diameter and were imaged with a 3D isotropic voxel size of 20 μm. Full 3D high-resolution raw data were obtained by rotating both the X-ray source and the flat panel detector 360° around the sample, with a rotation step of 0.1° (scanning time: 3 min). The corresponding 3600 image projections were then automatically reconstructed (RigakuSW software, Caliper) into a Dicom stack of 512 files using standard back-projection techniques (reconstruction time: less than a minute). The three-dimensional (3D) images of samples were built by stacking 512 cross sections obtained by X-ray microtomography. The resulting 3D files were composed of grey-level images where lowest grey/dark pixels represented empty spaces and highest grey/bright pixels stood for the densest materials. Three-dimensional analyses were performed using eXplore MicroView® software (General Electric Healthcare, Milwaukee, WI). Reconstruction of the region of interest was performed. Bone volume (BV) and total volume (TV) volume were measured for each group.

### Histological observation

Fixed samples in 4% (w/v) paraformaldehyde (PFA) were decalcified (Decalcifiant DC1, Labonord, France), dehydrated and paraffin-embedded. Transversal sections (7–9 µm thickness) were prepared and treated with Masson Trichrome for mineralized bone and osteoid tissue staining. The sections were analyzed with an Eclipse 80i light microscope (Nikon, Japan). Pictures were captured using a DXM 1200 C (Nikon, Japan) CCD camera.

Quantification of the bone formation was performed by measuring the amount of voxels containing collagen matrix of matured bone within the defects. For this purpose, using a Matlab script entitled ≪ L*a*b* Color Space ≫, a mask was drawn to encircle each defect within the bone. Then, mature bone was detected through a reference color value manually determined by the operator. Based on this reference, the script automatically attributed or not each pixel to this referenced color. Then, the number of voxels corresponding to collagen matrix of mature bone was calculated. This amount was normalized to the total amount of voxels covering the bone defect. This procedure was applied on 3 slides at each time point and for each biomaterials.

### Immunohistochemistry analysis

Immunohistochemistry for CD31 was performed as previously described^34^. Briefly, 7–9 µm thickness paraffin sections were cut, deparafinized, rehydrated using ethanol gradients and placed in PBS. Antigen recovery was performed using a proteinase K (Roche, France) digestion procedure and then endogenous peroxidase was quenched using 3% (v/v) hydrogen peroxide for 5 min at room temperature (RT). Tissues were blocked with 2% horse serum, in PBS, for 30 min at RT. Primary antibody against CD31 (NB100–2284, Novus Biologicals, Bio-Techne, France) was used at 1:100 in 2% (w/v) BSA in PBS, and incubated overnight at 4 °C. After two washes with PBS, the anti rabbit Impress kit (VectorLabs, USA) was used according to the manufacturer’s instructions. Specific staining was obtained using the 3,3′-diaminobenzidine staining solution (VectorLabs, USA). Counterstaining was performed using Mayer’s haematoxylin. Samples were then mounted using Pertex medium (Sigma). Sample imaging was performed using a microscope (Nikon Eclipse 80i) equipped with a digital camera (Nikon Dxm 1200C). Sample analysis was performed at three different sample positions and a total of six animal samples were assessed per time point and condition. Vessel quantification was performed using ImageJ analysis, and the number of vessels was determined and normalized to the area of the defect.

### Statistical Analysis

The Graphpad Prism 5.0 software was used. A D’Agostino and Pearson omnibus normality test was used in order to test if data obeyed to a Gaussian distribution. Statistically significant differences between several groups were analyzed by the one-way ANOVA test followed by the Bonferroni post test. A *p* value lower than 0.05 was considered to be statistically significant.

## Electronic supplementary material

Supplementary Information
