# Supplementary material for: 3D anatomical and perfusion MRI for longitudinal evaluation of biomaterials for bone regeneration of femoral bone defect in rats
Source: Sci Rep. 2017 Jul 21;7:6100. doi: 10.1038/s41598-017-06258-0 (PMC5522444; doi:10.1038/s41598-017-06258-0)
Supplement: Supplementary file 1 — Supplementary Information [file 41598_2017_6258_MOESM1_ESM.pdf]

3D anatomical and perfusion MRI for longitudinal evaluation of biomaterials for bone regeneration of femoral bone defect in rats

Emeline J Ribot[1*#], Clement Tournier[2#], Rachida Aid-Launais[3], Neha Koonjoo[1], Hugo Oliveira[2], Aurelien J Trotier[1], Sylvie Rey[2], Didier Wecker[4], Didier Letourneur[3], Joelle Amedee Vilamitjana[2], Sylvain Miraux[1]

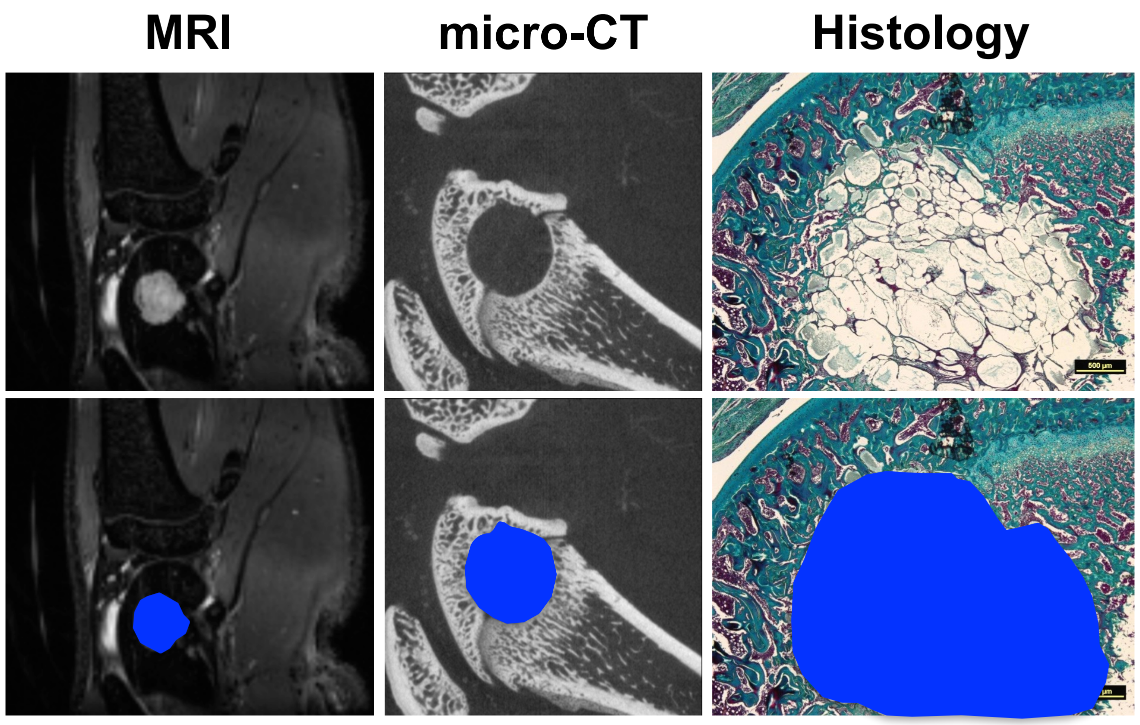

Supplementary Figure 1: Typical MRI, micro-CT and histology images at week 1 after implantation of a bone defect filled with a Matrix-HA-Fuco. The lower raw shows the biomaterial-filled defect area in blue on each modality.

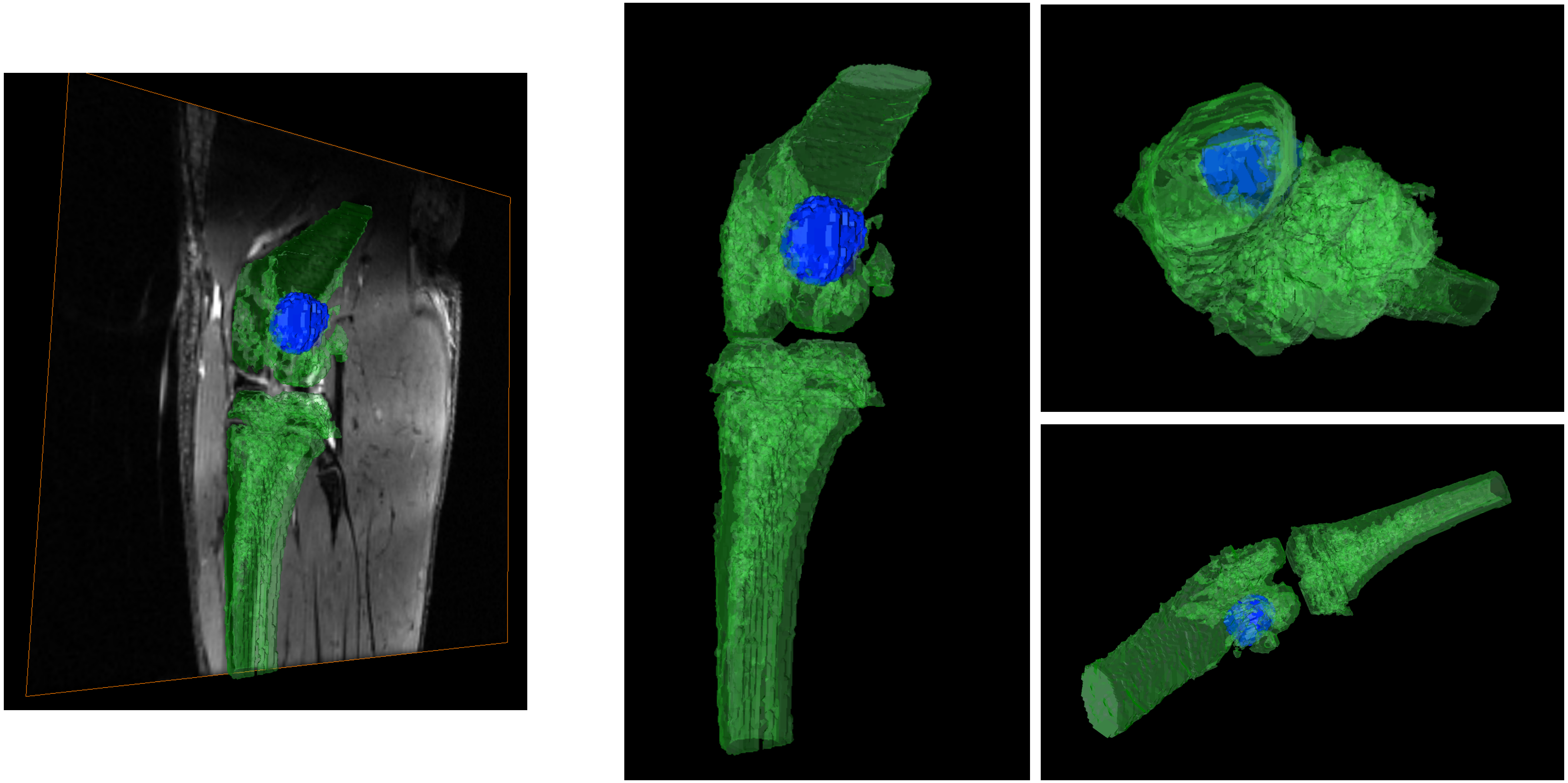

Supplementary Figure 2: 3D MRI representation of a rat knee with a femoral defect filled with a Matrix-HA-Fuco biomaterial, 1 week after implantation. On the left, extracted plan from a 3D MRI WS-bSSFP image and 3D super-imposed representation of the femoral and tibial bones (green) and the biomaterial (blue). On the right, 3D representations shown along 3 orientations.
